# Primary pancreatic hydatid cyst: a case report and literature review

**DOI:** 10.1186/s12876-021-01753-1

**Published:** 2021-04-13

**Authors:** Yilei Wu, Jun Gong, Wei Xiong, Xiaojiong Yu, Xiangyu Lu

**Affiliations:** 1 Department of Medical Records Statistics, Sichuan Provincial People’s Hospital, University of Electronic Science and Technology of China, Chengdu, 611731 Sichuan China; 2 The Second Department of Hepatobiliary Surgery, Sichuan Provincial People’s Hospital, University of Electronic Science and Technology of China, Chengdu, 611731 Sichuan China; 3 Chinese Academy of Sciences Sichuan Translational Medicine Research Hospital, Chengdu, 610072 Sichuan China

**Keywords:** Pancreas, Hydatid cyst, Diagnosis, Treatment, Case report

## Abstract

**Background:**

Hydatid cysts are parasitic zoonoses that often occur in the liver. Pancreatic hydatid cysts are very rare and are usually misdiagnosed as pancreatic cystadenomas. At present, surgical resection combined with albendazole administration is the standard treatment for pancreatic hydatid cysts. However, making accurate preoperative diagnoses and avoiding intraoperative cystic rupture are challenges for surgeons.

**Case presentation:**

A 28-year-old woman from the pastoral area presented to the surgical office complaining of abdominal pain and new-onset jaundice that began 9 days earlier. An enhanced computed tomography scan demonstrated a 6.0 × 5.3 cm pancreatic head cystic mass that compressed the common bile duct and induced choledochectasia. The preoperative diagnosis was pancreatic head cystadenoma, and laparotomic pancreaticoduodenectomy was initiated successfully. The intra- and postoperative diagnosis was pancreatic hydatid cyst. The patient was discharged uneventfully 7 days after the operation. A 1-year course of albendazole (15 mg/kg/day) was admitted.

**Conclusion:**

Pancreatic hydatid cysts are rare and often misdiagnosed as other types of cysts. History of living in an area in which the causative organism is endemic and positive anti-echinococcus IgG antibody status could help with the diagnosis. Radical resection combined with oral albendazole administration is the standard treatment for pancreatic hydatid cysts. Avoiding perioperative cystic rupture and abdominal echinococcosis implantation metastasis is crucial for the success of the operation.

## Background

Hydatid disease is a cosmopolitan zoonosis caused by the larvae of Echinococcus granulosus [1]. Hydatid disease is observed in the Northern Hemisphere, especially in China, the Russian Federation, continental European countries and North America [2]. Hydatid cysts often occur in the liver [3], and some extrahepatic organs including a rare form in the pancreas [4]. At present, some case reports of primary pancreatic hydatid cysts have been published [5–7]. However, the diagnosis is challenging due to its low morbidity, and the surgical procedure varies because of the different locations of the lesions and the understanding of this disease. In this article, we present a primary pancreatic hydatid disease case report and review published primary pancreatic hydatid cyst reports for better diagnosis and management.

## Case presentation

### Patient information

**Fig. 1:**
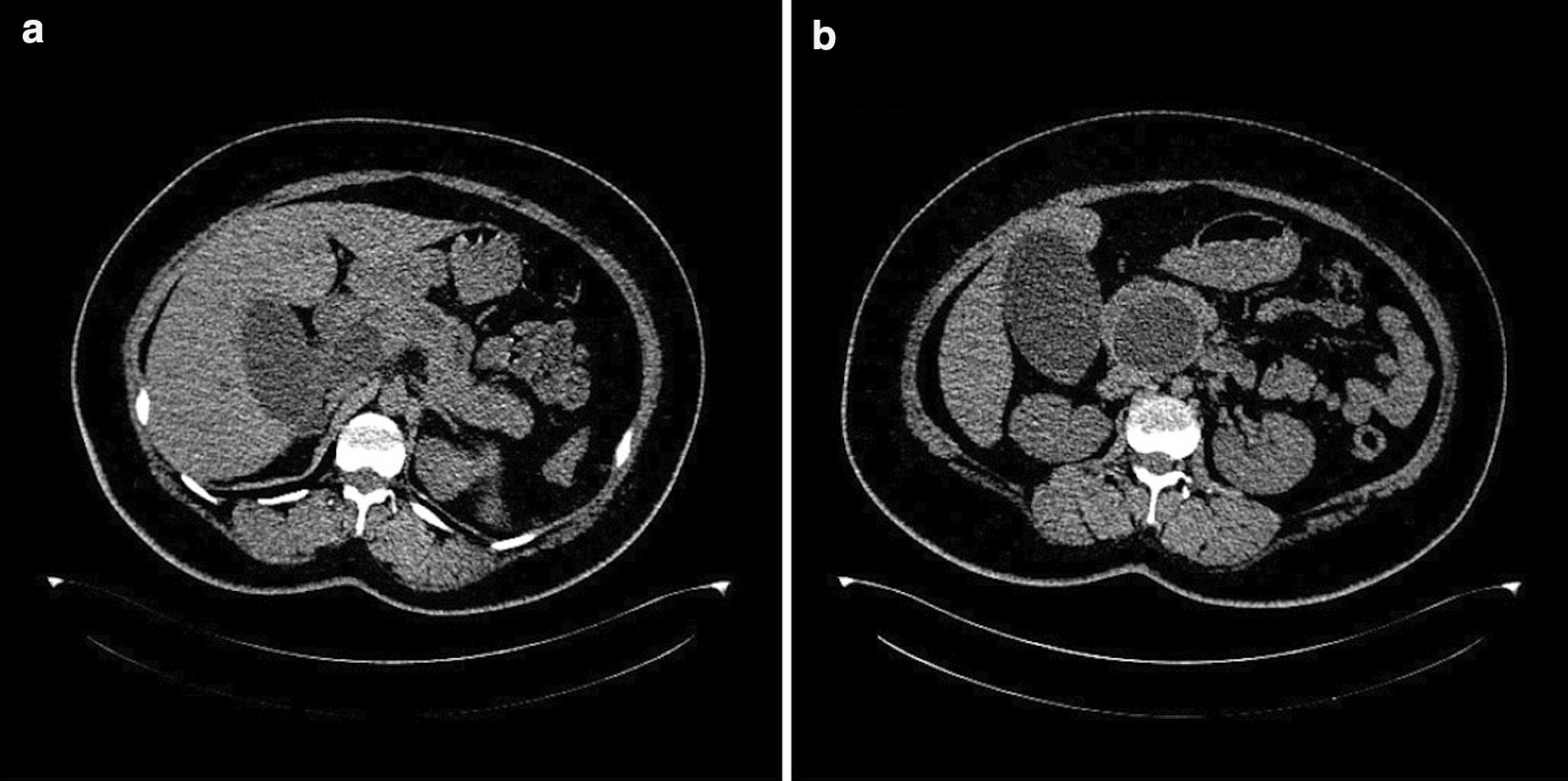
CT scans of the pancreatic hydatid cyst. **a** pancreatic cyst inducing bile duct and main pancreatic duct expanding; **b** pancreatic head hydatid cyst

**Fig. 2:**
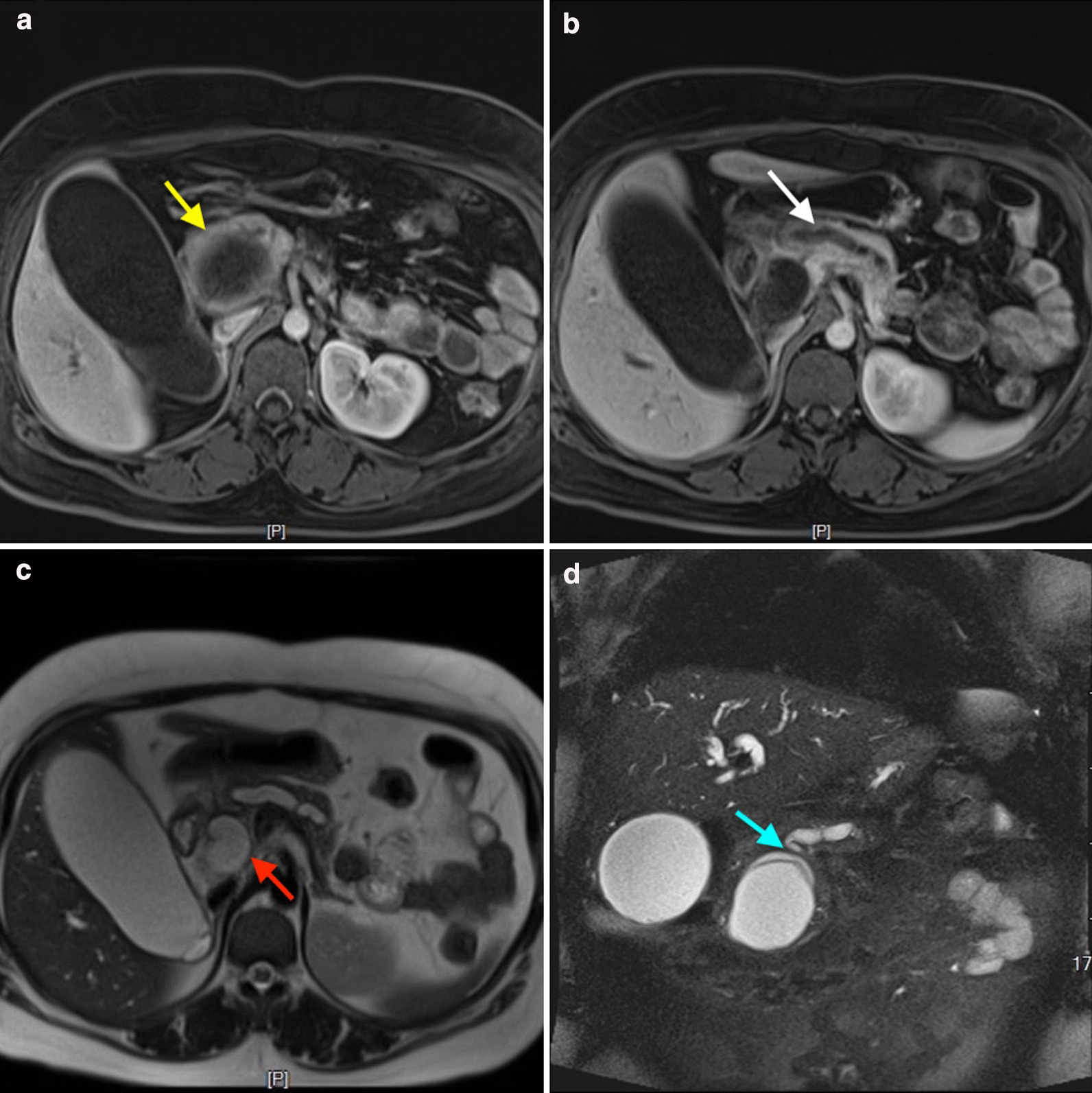
MRI of the pancreatic hydatid cyst. **a** The hydatid cyst mass (yellow arrow); **b** expanded main pancreatic duct (white arrow); **c** expanded bile duct (red arrow); **d** the hydatid cyst invaded the main pancreatic duct (blue arrow)

A 28-year-old woman presented to our department with body itching for more than ten days and jaundice for nine days. This patient had a long-term history of living in an area in which echinococcosis is endemic. Medical examination demonstrated yellow discoloration in both the skin and sclera, and abdominal examination was negative. After admission, the laboratory tests indicated a serum total bilirubin (Tbil) level of 264.0 μmol/L (5–22 μmol/L), conjugated bilirubin (Dbil) level of 205.6 μmol/L (0–7 μmol/L) and unconjugated bilirubin (Ibil) level of 58.4 μmol/L (0–20 μmol/L). Serum tumor markers, including carbohydrate antigen 19–9, α-fetoprotein and carcinoembryonic antigen, were not present. On the imaging examination, an emergency computed tomography (CT) scan indicated a 6.0 × 5.3 cm cystic lesion in the head of the pancreas adjacent to the lower bile duct and main pancreatic duct that induced expansion. There were no solid nodules or calcifications in this lesion, and a slightly higher lesion density with a curved mild calcification was observed at the edge of the cyst (Fig. 1). Magnetic resonance imaging (MRI) indicated the same signals at the center of the lesion and a high signal around the wall of the capsule, suggesting local diffusion limitation. MRCP imaging demonstrated that the bile duct and main pancreatic duct were clearly dilated, and the cyst communicated with the pancreatic bile duct (Fig. 2). Based on the preoperative examination, a diagnosis of pancreatic cystadenoma was made.

### Surgical procedure

**Fig. 3:**
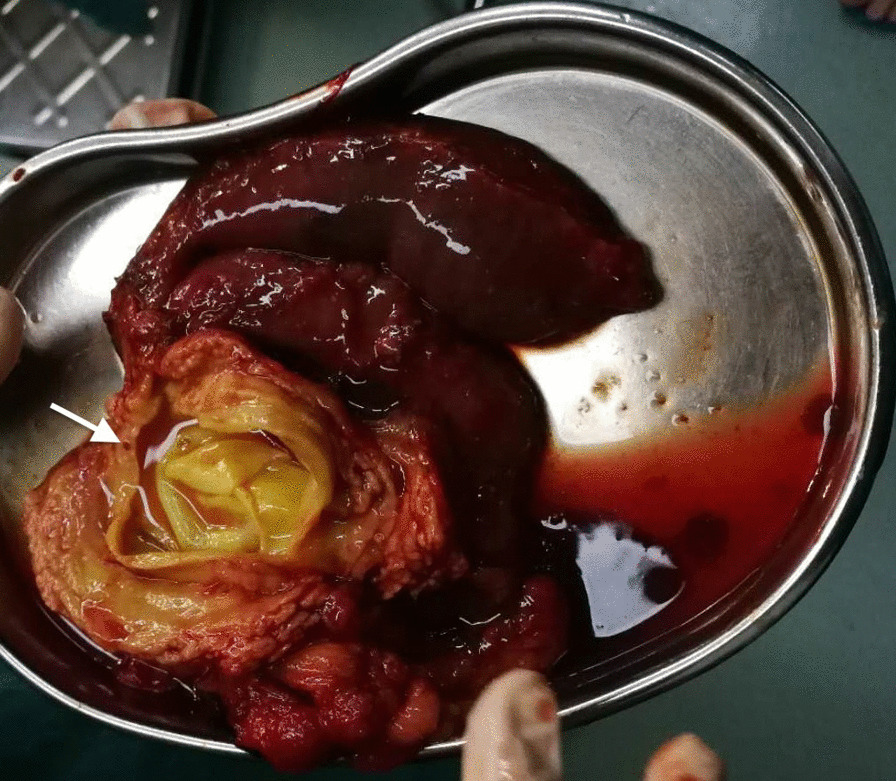
The resected mass. The main pancreatic duct was invaded (white arrow)

**Fig. 4:**
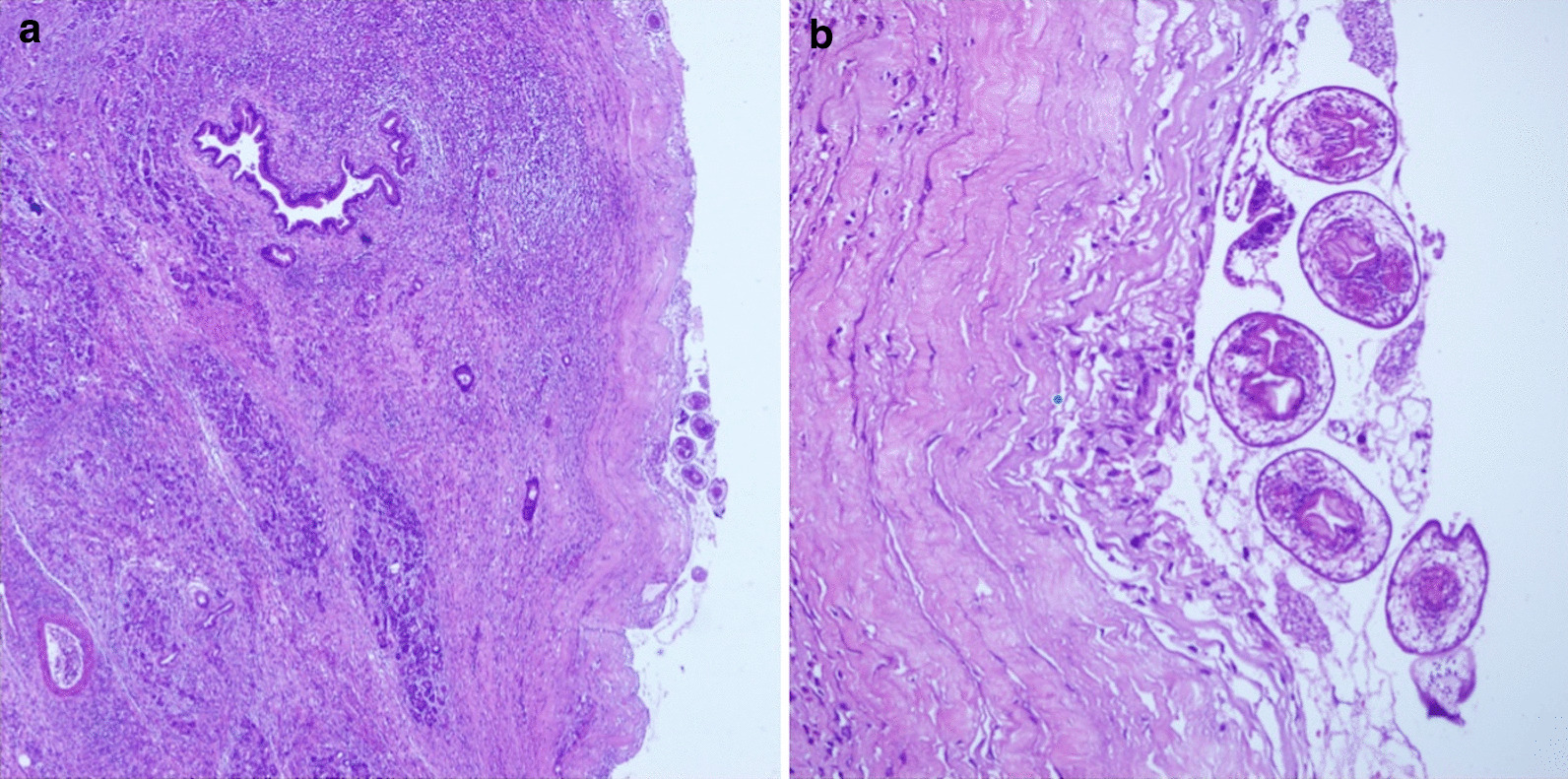
Pathological examination of the pancreatic hydatid cyst: **a** ×40; **b** ×200

During the laparotomy, a cyst lesion located in the pancreatic head was detected, which was adjacent to the duodenum and invaded the main pancreatic duct. After the cyst fluid was completely aspirated, the internal cyst collapsed and separated from what was previously considered the cyst wall (Fig. 3). The intraoperative pictures demonstrated signs of hydatid cysts (CE-1). The pancreaticoduodenectomy procedure was initiated successfully, and a “Child type” chongio-/gastric/pancreatic/jejunal anastomosis was performed. The patient was discharged uneventfully 7 days after the operation. A 1-year course of albendazole (15 mg/kg/day) was admitted and no recrudescence was detected in the postoperative 2 years. Pathological results indicated the presence of Echinococcosis granulosus accompanied by inflammatory cell infiltration and eosinophilic infiltration (Fig. 4).

### Literature review

A literature search was initiated to review primary pancreatic hydatid cysts. Based on our search results (Table 1), 22 papers in PubMed reported 33 cases of primary pancreatic hydatid cysts from 2011 to 2021 [8–29]. In the 33 cases, 14 cases had cysts located in the pancreatic head, and 15 cases had lesions in the body/tail. Nineteen cases reported serum IgG levels, whereas the results of 8 cases were negative.

**Table 1:** Literature review of the primary pancreatic hydatid cyst in PubMed (2011–2021)

First author [ref]	Case count	Sex (M/F)	Age (year)	Location	Cyst size (mm)	Serology	Surgical procedure
Tavusbay [18]	1	F	48	Head	28 × 25	Negative	Partial cystectomy + omentoplasty
Agrawal [10]	1	F	5	Head	120 × 110	NS	Enucleation + cholangiography + cholecystectomy + cystography
Bhat [29]	1	F	4	Head	100 × 150 × 70	Negative	Partial cystectomy + drainage
Masoodi [24]	1	M	45	Tail	70 × 60	Positive	Distal pancreatectomy + splenectomy
Varshney [16]	1	M	35	Tail	NS	Positive	Distal pancreatectomy
Suryawanshi [9]	1	M	20	Head	80 × 80	NS	Cyst evacuation + omentoplasty
Karaman [19]	1	M	32	Neck	55 × 45	Positive	Percutaneous drainage
Makni [12]	1	M	38	Tail + body	100 × 90	Positive	Distal pancreatectomy + splenectomy
Mandelia [22]	1	M	6	Head	54 × 41	NS	Enucleation + cholangiography
Yarlagadda [17]	1	M	43	Tail	180 × 170	NS	Distal pancreatectomy + splenectomy
Trigui [13]	12	F	21	Tail	NS	NS	Distal pancreatectomy
		M	13	Tail + body	NS	NS	Partial cystectomy + drainage
		M	15	Head	NS	NS	Partial cystectomy + drainage
		M	26	Head	NS	NS	Partial cystectomy + drainage
		F	50	Head	NS	NS	Partial cystectomy + drainage
		F	37	Head	25	Negative	Pancreaticoduodenectomy
		M	8	Head	83 × 76	Positive	Partial cystectomy + duodenal fistula treatment
		F	26	Tail + body	40	Positive	Distal pancreatectomy + splenectomy
		F	61	Tail	NS	Positive	Distal pancreatectomy + splenectomy
		F	11	Head	NS	Negative	Cysto-duodenal anastomosis
		F	16	Body	NS	Positive	Partial cystectomy + drainage
		F	11	Head	50	NS	Cysto-duodenal anastomosis
Vasilescu [27]	1	F	63	Isthmus + body	110 × 86	Negative	Drainage + lavage with hypertonic serum + partial cystectomy
Deak [23]	1	M	34	Head	60 × 40 × 40	Negative	Enucleation + partial cystectomy
Alsaid [20]	1	M	34	Body	350 × 200 × 150	NS	Partial cystectomy + drainage
Bakkaly [14]	1	F	5	Head	57 × 31	Positive	Partial cystectomy + drainage
Sethi [8]	1	F	48	Tail + body	110 × 140	Negative	NS
Lada [26]	1	F	18	NS	120 × 130 × 110	Negative	Partial cystectomy + drainage
Ahmed [21]	1	F	40	Body	55 × 57	Positive	Partial cystectomy + drainage
Elaffand [15]	1	M	34	Body + tail	110 × 80	Positive	Partial cystectomy + cystogastrostomy
Hiremath [11]	1	F	48	Neck	80 × 100 × 82	NS	Partial cystectomy + drainage
Kisaoglu [28]	1	F	58	NS	50 × 30	NS	Splenectomy + distal pancreatectomy + cholecystectomy
Jonkov [25]	1	F	61	Body + tail	55 × 75	NS	Distal pancreatectomy

*NS* not-stated

## Discussion and conclusion

Primary pancreatic hydatid cysts account for 0.14–2% of the total number of systemic echinococcoses [21]. Primary pancreatic hydatid cysts are caused by the Echinococcus granulosus hookworm entering the systemic circulation and traveling to the pancreas through two barriers, including the liver and lung. Primary pancreatic hydatid cysts often occur in the pancreatic head rather than body/tail because of richer blood supply for the pancreatic head [26]. However, there are 12 cases occurred in the pancreatic head and 12 cases occurred in the pancreatic body/tail in a total of 33 cases based on the literature review. The reason for the comparable occurring rate is the limited reviewed manuscripts.

There are no specific clinical signs when the cyst is located in the pancreatic body/tail, whereas obstructive jaundice may occur when the lesion is in the head of the pancreas, such as the presented case. Also, some cases may present acute pancreatitis firstly because of the main pancreatic duct obstruction [9, 12]. Because of its rare incidence, pancreatic hydatid cysts are often misdiagnosed as other cyst types, such as pancreatic cystadenoma or cystadenocarcinoma. For the cases with suspected pancreatic hydatid cysts, most had the characteristics of living in areas where echinococcosis is endemic and a history of close contact with sheep or dogs. These characteristics and imaging data should be combined to diagnose echinococcosis.

Enhancing CT scans, MRI and/or contrast-enhanced ultrasound examinations facilitate diagnosis. The characteristics of signals or multiple subcysts, calcifications and consolidation may be present in the resulting images. Kerimoglu et al. demonstrated that mild enhancement of the medial edge of the capsule wall might occur [30]. In our case, the image of the pancreatic tail shows a single capsule with a clear boundary. The cross-section image shows local nodular thickening of the cystic wall, and it is difficult to differentiate hydatid cysts from other cystic lesions of the pancreas. Also, contrast-enhanced ultrasound examinations may help for the identification because the contrast medium could enter the internal septations of cystadenoma or cystadenocarcinoma, whereas the cystic echinococcosis presented non-contrast medium entered in the sub-cysts, and the sub-cysts could move with the patient’s position changing [31]. Serum-positive anti-echinococcal IgG antibody status could help with the diagnosis, whereas which may be present due to the patient coming into contact with Echinococcus while living in an endemic area, rather than the assumed pancreatic hydatid cyst. In the reviewed literature, there are only 11 cases with positive anti-echinococcus IgG antibody in the 19 cases of confirmed pancreatic hydatid cyst.

Several surgical procedures have been described for the treatment of pancreatic hydatid cysts depending on their location [32]. Presently, available treatment options include radical resection, internal capsule stripping and external capsule removal (subadventitial total exocystectomy). In our case, the cyst was located in the pancreatic head, involved the main pancreatic duct and caused obstructive jaundice due to compression of the common bile duct; therefore, we performed pancreaticoduodenectomy. Based on the literature review, surgical drainage procedures have also been recommended but should not be the first selection because of the risk for abdominal intraperitoneal hydatid cyst implantation and metastasis. Additionally, cyst fluid overflow should be avoided during surgery.

In conclusion, pancreatic hydatidosis is a rare disease, and preoperative diagnosis of isolated pancreatic hydatid cysts in endemic regions is infrequent. It is difficult to differentiate between benign and malignant tumors of the pancreas before surgery. CT, MRI and/or contrast-enhanced ultrasound examination could be used to evaluate the malignant potential of the cyst and the presence of any connection with the main pancreatic duct. To clarify the relationship between cysts and the pancreatic duct, ERCP can be used to identify unknown cysts [33]. For cases strongly suspected to be pancreatic hydatids, preoperative evaluation should be combined with radiography and laboratory examination. A surgical procedure to prevent intra-abdominal spread with albendazole therapy thereafter may be an effective treatment for the disease.

## Data Availability

All data generated or analyzed during this study are included in this published article.

## Abbreviations

Tbil: Total bilirubin
Dbil: Conjugated bilirubin
Ibil: Unconjugated bilirubin
CT: Computed tomography
MRI: Magnetic resonance imaging
MRCP: Magnetic Resonance Cholangiopancreatography
ERCP: Endoscopic Retrograde Cholangio Pancreatography
